# Prevalence and Risk Factors of Postprandial Hypotension Among Japanese Older Adults in a Facility

**DOI:** 10.1093/geroni/igaa057.716

**Published:** 2020-12-16

**Authors:** Noriko Suzuki, Masahiko Hashizume, Hideyuki Shiotani

**Affiliations:** 1 Seisen Jogakuin College, Nagano-shi, Nagano-ken, Japan; 2 Arima Onsen Hospital, Kobe, Japan; 3 Kobe University, Kobe city, Hyogo, Japan

## Abstract

Postprandial hypotension (PPH) is an unrecognized sudden drop of blood pressure (BP) after meals and a hidden problem among older people including those living in long-term care facilities (LTCFs). Though PPH causes dizziness, falls, and syncope, it has received little attention from¬¬¬ healthcare workers (HCW) including caregivers, nurses and physicians, and risk factors of PPH should be carefully assessed to improve quality of life. Therefore, we aimed to examine the prevalence and risk factors of PPH in a LTCF in Japan. Participants were 114 older adults living in a LTCF in Japan (mean age 85.9 years old; 85 female (74%)). To examine PPH, blood pressure (BP) was measured before and after lunch. BP after meal was measured four times every 30 minutes. PPH is defined as a BP drop of 20 mmHg or more and we also defined a BP drop within a range of 19 to 15 mmHg as potential-PPH. As risk factors, we compared systolic and diastolic BP at baseline, body mass index, pulse rate, disease and complications between groups with/without PPH. The prevalence of PPH was 41% (47/114) and 52% with potential-PPH; 11% (13/114) added. Among risk factors, systolic BP was significantly higher in those with PPH (142.6 vs 123.5 mmHg, p <0.001). This study revealed that PPH & potential-PPH occurred in half of the subjects in a LTCF in Japan. HCW need to focus on high systolic BP to predict PPH and future research is necessary to prevent and cope with PPH for older people.

